# Peptide encoded by lncRNA BVES-AS1 promotes cell viability, migration, and invasion in colorectal cancer cells via the SRC/mTOR signaling pathway

**DOI:** 10.1371/journal.pone.0287133

**Published:** 2023-06-22

**Authors:** Weiwei Zheng, Yingchang Guo, Guangtan Zhang, Junwei Bai, Yucheng Song, Xiaofei Song, Qinhui Zhu, Xuebin Bao, Gang Wu, Chao Zhang

**Affiliations:** 1 Department of Gastrointestinal Surgery, Henan Provincial People’s Hospital, People’s Hospital of Zhengzhou University, School of Clinical Medicine, Henan University, Zhengzhou, Henan Province, China; 2 Department of Interventional Therapy, the First Affiliated Hospital of Xinxiang Medical College, Xinxiang, Henan Province, China; 3 Department of General Surgery, Shangcai People’s Hospital, Zhumadian, Henan Province, China; BMSCE: BMS College of Engineering, INDIA

## Abstract

Long non-coding RNAs (lncRNAs) have been revealed to harbor open reading frames (ORFs) that can be translated into small peptides. The peptides may participate in the pathogenesis of colorectal cancer (CRC). Herein, we investigated the role of a lncRNA BVES-AS1-encoded peptide in colorectal tumorigenesis. Through bioinformatic analysis, lncRNA BVES-AS1 was predicted to have encoding potential and to be associated with poor prognosis of patients with CRC. In CRC cells, BVES-AS1 was validated to encode a 50-aa-length micro-peptide, named BVES-AS1-201-50aa, through a western blotting method. BVES-AS1-201-50aa enhanced cell viability and promoted the migratory and invasive capacities of HCT116 and SW480 CRC cells *in vitro*, validated via CCK-8 assay and transwell assay, respectively. Immunofluorescence assay showed that BVES-AS1-201-50aa increased the expression of proliferating cell nuclear antigen (PCNA) and matrix metalloproteinase 9 (MMP9) in CRC cells. We further verified that BVES-AS1-201-50aa targeted and activated the Src/mTOR signaling pathway in CRC cells by co-immunoprecipitation (Co-IP) experiment, qualitative proteomic analysis, and western blotting. Our findings demonstrated that BVES-AS1 could encode a micro-peptide, which promoted CRC cell viability, migration, and invasion *in vitro*. Our current work broadens the diversity and breadth of lncRNAs in human carcinogenesis.

## Introduction

Colorectal cancer (CRC) is a malignant colorectal neoplasm all over the world and, despite intensive research and clinical efforts, remains the second leading cause of cancer related mortality [1, 2]. In 2020, more than 1,900,000 new CRC cases and 935,000 deaths are estimated globally [1]. Additionally, CRC has an incidence of 252.96 in 100,000 people and a high death of 169.27 per 100,000 people in adults over 75 years old in 2019 worldwide [3]. Moreover, with the increasing trend in younger-onset CRC in Chinese patients, the burden of CRC is on the rise in China [4]. Thus, further research is needed to uncover molecular mechanisms underlying CRC pathogenesis for development of novel targeted therapies. In recent years, the integral role of non-coding transcripts in colorectal tumorigenesis and CRC progression has been documented [5]. For example, numerous studies have revealed that many long non-coding RNAs (lncRNAs) regulate CRC-related malignant processes by operating as oncogenic drivers or anti-tumor factors [6–8].

Lately, it has become evident that a number of lncRNAs can be actually translated into peptides and thus contributes to human carcinogenesis [9, 10]. Although lncRNAs were initially defined as non-coding transcripts without encoding potential, the advancement of ribosome profiling and sequencing technologies has revealed that some lncRNAs harbor short open reading frames (ORFs) that give them coding activity [11]. Indeed, several peptides encoded by lncRNAs have been implicated in the regulation of the malignant behaviors of CRC cells [12, 13]. For instance, MEK1-binding onco-peptide (MBOP), an 85-amino acid (aa) micro-peptide that is encoded by LINC01234, is highly expressed in CRC and drives the motility and growth of CRC cells via the MEK1/pERK/MMP2/MMP9 regulatory cascade [14]. ATP synthase-associated peptide (ASAP) is a small 94-aa peptide encoded by LINC00467, and it is capacity of enhancing CRC cell proliferation by elevating the activity of ATP synthase [15]. Moreover, the LOC90024-encoded peptide splicing regulatory small protein (SRSP) is associated with a poor prognosis of CRC patients and can function as a promoter in increasing CRC cell growth, motility, and invasion [16].

Src, a protein tyrosine kinase that involves the regulation of cellular survival, motility, angiogenesis, and growth, is a well-known proto-oncogene implicated in cancer biology [17]. Src can also affect cancer cell metabolism, such as glycolysis and oxidative phosphorylation [18], and targeting Src has been proposed as a promising approach in cancer treatment [17]. High expression of Src in patients with CRC predicts poor survival outcomes [19] and can contribute to CRC cell malignant phenotypes, including growth and metastasis [20]. Although Src has been identified as a pivotal mediator of cellular pro-tumorigenic signals in CRC [21, 22], our understanding of its upstream regulators remains limited.

Here, we elucidate that BVES-AS1, annotated as a lncRNA in humans that is aberrant expression in CRC [23], can encode a 50-aa-length micro-peptide (named BVES-AS1-201-50aa) in HCT116 and SW480 CRC cells. Consequently, we explored the activity and mechanism of the short peptide in CRC pathogenesis and demonstrated the promoting effect of BVES-AS1-201-50aa on CRC cell growth, migration, and invasion. Hence, BVES-AS1-201-50aa might function as a potential target against CRC.

## Materials and methods

### Bioinformatics

The online database GEPIA (http://gepia2.cancer-pku.cn/#index) was utilized to search the lncRNAs that are differentially expressed in CRC specimens and that are associated with poor prognosis of patients with CRC. To find the lncRNAs with coding potential, we used Translnc online software (http://bio-bigdata.hrbmu.edu.cn/TransLnc/download.jsp), which was also used to download the detailed information of lncRNAs-encoded peptides. The GO enrichment analysis and KEGG pathway enrichment analysis were performed on DAVID database (https://ngdc.cncb.ac.cn/databasecommons/database/id/3061). The protein-protein interaction (PPI) network was analyzed on the online tool String (https://cn.string-db.org/).

### Cell lines

The two CRC cell lines HCT116 and SW480 (Procell, Wuhan, China) used in our study were maintained in DMEM (Servicebio, Wuhan, China) containing 10% heat-inactivated FBS (Servicebio), enriched with 1% penicillin/streptomycin (Solarbio, Beijing, China). Cells were routinely maintained at 37°C, 5% CO_2_ and 85% humidity.

### Plasmids and transient transfection of cell lines

To confirm the encoding property of the seven lncRNA-hidden ORFs (S1 Table), the corresponding DNA sequences were synthesized and cloned into pcDNA3.1–3×Flag plasmid individually, which was performed by genecreate (Wuhan, China). We named the pcDNA3.1-Flag-BVES-AS1-201-50aa plasmid as ORFwt-Flag (or ORF-wt). The plasmid constructs pcDNA3.1-GFP-BVES-AS1-201-50aa (ORFwt-GFP) and the mutations (ORFmut-Flag and ORFmut-GFP), in which the start codon ATG was mutated to ATT, were obtained from Tsingke (Beijing, China). As controls, the nontarget plasmids pcDNA3.1-Flag (Vector) and pcDNA3.1-GFP (Control) were used.

HCT116 and SW480 CRC cells were plated in 6-well culture dishes at 1.0 × 10^5^ cells/well. On the next day, transient introduction was performed by transfecting 3 μg of plasmids into the cells using 5 μL RFect Plasmid DNA Transfection Reagent as described by the manufacturer (Baidai, Changzhou, China). The transfected cells were harvested 12 h later for the subsequent function assays or subjected to expression analysis 24 h later.

### Western blotting

For immunoblot analyses, 1.0 × 10^7^ transfected cells were lysed (0°C; 30 min) in 100–200 μL of 1× RIPA buffer (Solarbio) containing a Phosphatase & Protease inhibitor cocktail (Solarbio). Approximately 20 μg of protein samples was loaded on SDS polyacrylamide gels and subjected to electrophoresis prior to transfer to nitrocellulose membranes (Bio-Rad, Sundbyberg, Sweden). Probing was conducted using specific antibodies to Flag (mouse monoclonal, Cat#80010-1-RR, 1:1000, Proteintech, Wuhan, China), Src (mouse monoclonal, Cat#60315-1-Ig, 1:20000, Proteintech), phosphorylated-Src (p-Src, Tyr416, rabbit monoclonal, Cat#59548S, 1:1000, Cell Signaling Technology, Beverly, MA, USA), mTOR (mouse monoclonal, Cat#66888-1-Ig, 1:10000, Proteintech), p-mTOR (Ser2448, mouse monoclonal, Cat#67778-1-Ig, 1:5000, Proteintech), and GAPDH (mouse monoclonal, Cat#60004-1-lg, 1:50000, Proteintech). The goat anti-mouse or anti-rabbit IgG coupled by HRP (Cat#SA00001-1 or Cat#SA00001-2, 1:5000, Proteintech) served as the secondary antibody. After visualization with enhanced chemiluminescence (Yeasen, Shanghai, China), immunoreactivity signals were analyzed using the Chemidoc-XRS Gel System with Quantity One software (Bio-Rad).

### *In vitro* viability assay (CCK-8)

HCT116 and SW480 CRC cells after transient transfection were seeded in triplicates at 2 × 10^3^ cells/well in 96-well culture dishes. At 48 h after plating, cells were carried out by CCK-8 assay as per the manufacturing instructions (Beyotime, Shanghai, China). After addition of the CCK-8 reagent (10 μL/well), the incubation was performed for 2 h at 37°C, followed by the measurement of the absorbance at 450 nm with a plate reader (BioTek, Winooski, VT, USA).

### Immunofluorescence

After being fixed in 4% paraformaldehyde for 15 min, transfected CRC cells were permeabilized for 20 min with Triton X-100 (0.5%) and blocked for 30 min with BSA (3%, Beyotime). Then, cells were probed with rabbit polyclonal anti-matrix metalloproteinase 9 (anti-MMP9, Cat#GB11010, 1:500, Servicebio) or anti-proliferating cell nuclear antigen (anti-PCNA, Cat#GB11132, 1:1000, Servicebio) antibody overnight at 4°C before the incubation with goat anti-rabbit IgG secondary antibody conjugated by Alexa Fluor 488 (Cat#GB25303, Servicebio) for 1 h at 37°C in the dark. DAPI was subsequently used for nuclear staining. Fluorescence images were obtained under a fluorescence microscope (BX53, Olympus, Tokyo, Japan), and data analysis were done with ImageJ software (NIH, Bethesda, MA, USA).

### Transwell migration and Matrigel invasion assays

For invasion analysis, transfected CRC cells were plated in a Matrigel-coated chamber with 8.0 μm pore size membranes (Corning Costar, Corning, NY, USA). For motility analysis, transfected CRC cells were seeded atop uncoated membranes with 8.0 μm pore size (Corning Costar). In both assays, cells (1.0 × 10^5^/well) in non-serum DMEM were translocated toward complete growth medium for 24 h. After that, 0.1% crystal violet (Servicebio) was used for the staining of the cells that had invaded or migrated to the lower chamber. Pictures were taken under the microscope (Olympus), and the stained cells were counted in at least 5 random fields from each sample.

### Co-immunoprecipitation (Co-IP) experiments

Vector- and ORF-wt-transfected HCT116 cells were lysed (0°C; 30 min) with RIPA buffer containing a Phosphatase & Protease inhibitor cocktail. Meantime, the mixture of sepharose A/agarose G beads (Thermo Fisher Scientific, Saint-Aubin, France) and antibodies against Flag (Cat#80010-1-RR, 1.0 μg) or isotype IgG (Cat#30000-0-AP, 1.0 μg, Proteintech) was made and incubation was performed for 2 h at 4°C. Then, whole-cell lysates were incubated with the mixture for 4 h at 4°C. Beads were subsequently harvested and washed three times with the same buffer and proteins bound to beads were eluted with the protein eluent.

### Silver staining and qualitative proteomic analysis

The precipitated proteins in Co-IP experiments were subjected to SDS-PAGE prior to silver staining (for visualization of proteins) as previously reported [24]. Briefly, after SDS-PAGE, the gels were fixed in fixation solution (10% acetic acid and 40% ethanol) for 30 min, incubated with protein treatment solution for 30 min, and washed with 0.5% dichromate. The gels were equilibrated with 0.1% silver nitrate solution (Kaidac, Baoji, China) for 30 min at room temperature and then stained with complex formation solution (0.02% paraformaldehyde and 3% Na_2_CO_3_). After being incubated with stop solution, the gels were fixed onto a polyester sheet (Bio-Rad) for indefinite storage.

The qualitative proteomic analysis of the precipitated proteins was carried out by Qinglianbio Biotechnology Co., Ltd. (Beijing, China) based on the HPLC-MS/MS method using RIGOL L-3000 HPLC System (RIGOL, Beijing, China), and the data were analyzed by Proteome Discoverer2.4 software (Thermo Fisher Scientific).

### Statistical analysis

Data were presented as mean ± SD of separate experiments (n ≥ 3). Differences were compared by GraphPad Prism 8 (GraphPad Inc., La Jolla, CA, USA) using Tukey’s multiple comparisons test after one-way or two-way ANOVA. Statistical significance for all the tests, evaluated by calculating *P* value, was < 0.05.

## Results

### BVES-AS1 encodes a short peptide BVES-AS1-201-50aa

**Fig 1:**
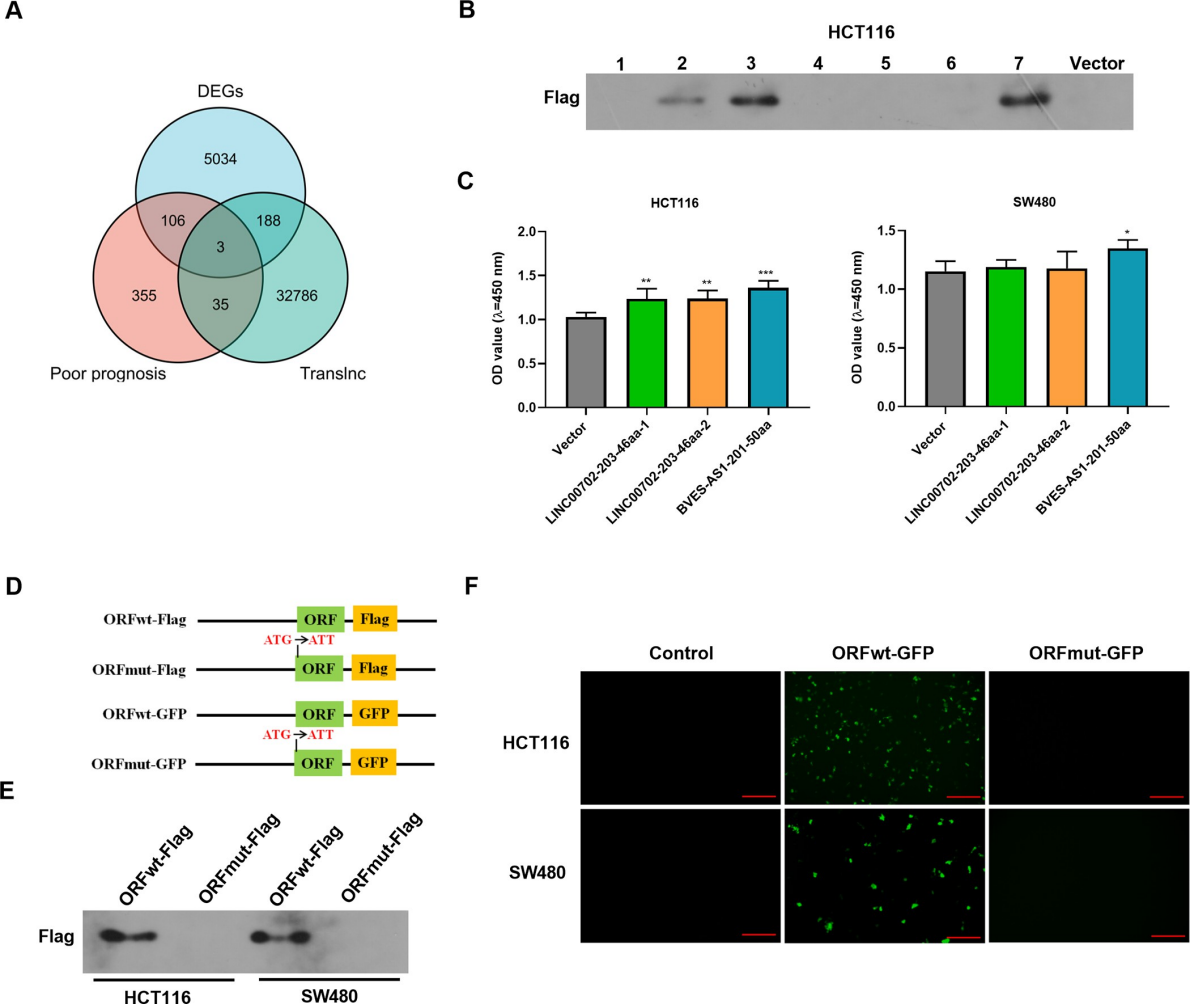
Screen and identification of BVES-AS1-201-50aa. (A) Venn diagram showed the lncRNAs that are aberrant expression in CRC, are associated with poor prognosis of CRC patients, and are predicted to have encoding potential. (B) Western blotting analysis measured the expression of three peptides (LINC00702-203-46aa-1, LINC00702-203-46aa-2 and BVES-AS1-201-50aa) in HCT116 cells using the anti-Flag antibody. The lanes were loaded as follows: line 1, ALOX12-AS1-207-17aa; line 2: LINC00702-203-46aa-1; line 3: LINC00702-203-46aa-2; line 4: LINC00702-201-25aa; line 5: BVES-AS1-202-21aa-1; line 6: BVES-AS1-202-21aa-2; line 7: BVES-AS1-201-50aa. (C) CCK-8 assay was used to assess the effect of the three peptides on cell viability. (D) Schematic diagram of the expression constructs fused with Flag tags or a GFP tag (ORFwt-Flag and ORFwt-GFP) and the mutations (ORFmut-Flag and ORFmut-GFP) in which the start codon ATG was mutated to ATT. (E) Western blotting showed the expression of BVES-AS1-201-50aa-Flag fusion protein of the two constructs fused with Flag tags in HCT116 and SW480 cells using the anti-Flag antibody. (F) Fluorescence images revealed the expression of BVES-AS1-201-50aa-GFP fusion protein of the two constructs fused with a GFP tag in HCT116 and SW480 cells. Scale Bar: 100 μm. *P<0.05, **P<0.01, ***P<0.001.

To observe deregulated lncRNAs implicated in CRC pathogenesis, we first analyzed the differentially expressed lncRNAs (DEGs) in CRC and the lncRNAs that are associated with poor prognosis of patients with CRC on the online database GEPIA (http://gepia2.cancer-pku.cn/#index). By combining the above CRC-related lncRNAs with lncRNAs that have encoding potential predicted by Translnc online software (http://bio-bigdata.hrbmu.edu.cn/TransLnc/download.jsp), a total of three lncRNAs (ALOX12-AS1, LINC00702 and BVES-AS1) were found, as illustrated by Venn diagram (Fig 1A). Based on the combination of the high prediction score of their encoding ability on Translnc database and the expression of their protein transcripts in CRC tumors on GEPIA database, seven peptide transcripts encoded by the three lncRNAs were found (S1 Table). To determine the encoding property of the seven transcripts, the corresponding DNA sequences were cloned into the pcDNA3.1 expression plasmid fused with 3× Flag tags, respectively, and then were transfected in HCT116 CRC cells. Through western blotting analysis with the anti-Flag antibody, three peptides (LINC00702-203-46aa-1, LINC00702-203-46aa-2 and BVES-AS1-201-50aa) were validated to be expressed in HCT116 cells (Fig 1B). The influence of the three peptides on viability of HCT116 and SW480 CRC cells was further investigated by CCK-8 assay. Interestingly, transfection of cells with the corresponding plasmids encoding the three peptides showed that only BVES-AS1-201-50aa expressed by ORFwt-Flag was able to promote the viability of both HCT116 and SW480 cell lines (Fig 1C). We thus focused on BVES-AS1-201-50aa in the subsequent study.

To demonstrate whether the start codon ATG of the BVES-AS1-201-50aa ORF is functional, the mutant construct (ORFmut-Flag) was generated, in which the start codon ATG was mutated to ATT (Fig 1D). The expression of BVES-AS1-201-50aa-Flag fusion peptide was observed in ORFwt-Flag-transfected CRC cells, but not ORFmut-Flag-introduced cells (Fig 1E), demonstrating the activity of the start codon of the BVES-AS1-201-50aa ORF in protein encoding. To further confirm the finding, the wild-type and mutant expression plasmids (ORFwt-GFP and ORFmut-GFP) fused with GFP tag were constructed, as seen in Fig 1D. The BVES-AS1-201-50aa-GFP fusion peptide was detected in ORFwt-GFP-transfected cells, and the mutation in the start codon abolished the expression of the GFP fusion peptide (Fig 1F). Collectively, these data demonstrate that BVES-AS1 can encode a short peptide BVES-AS1-201-50aa.

### BVES-AS1-201-50aa promotes the growth and enhances the migratory and invasive potential of CRC cells

**Fig 2:**
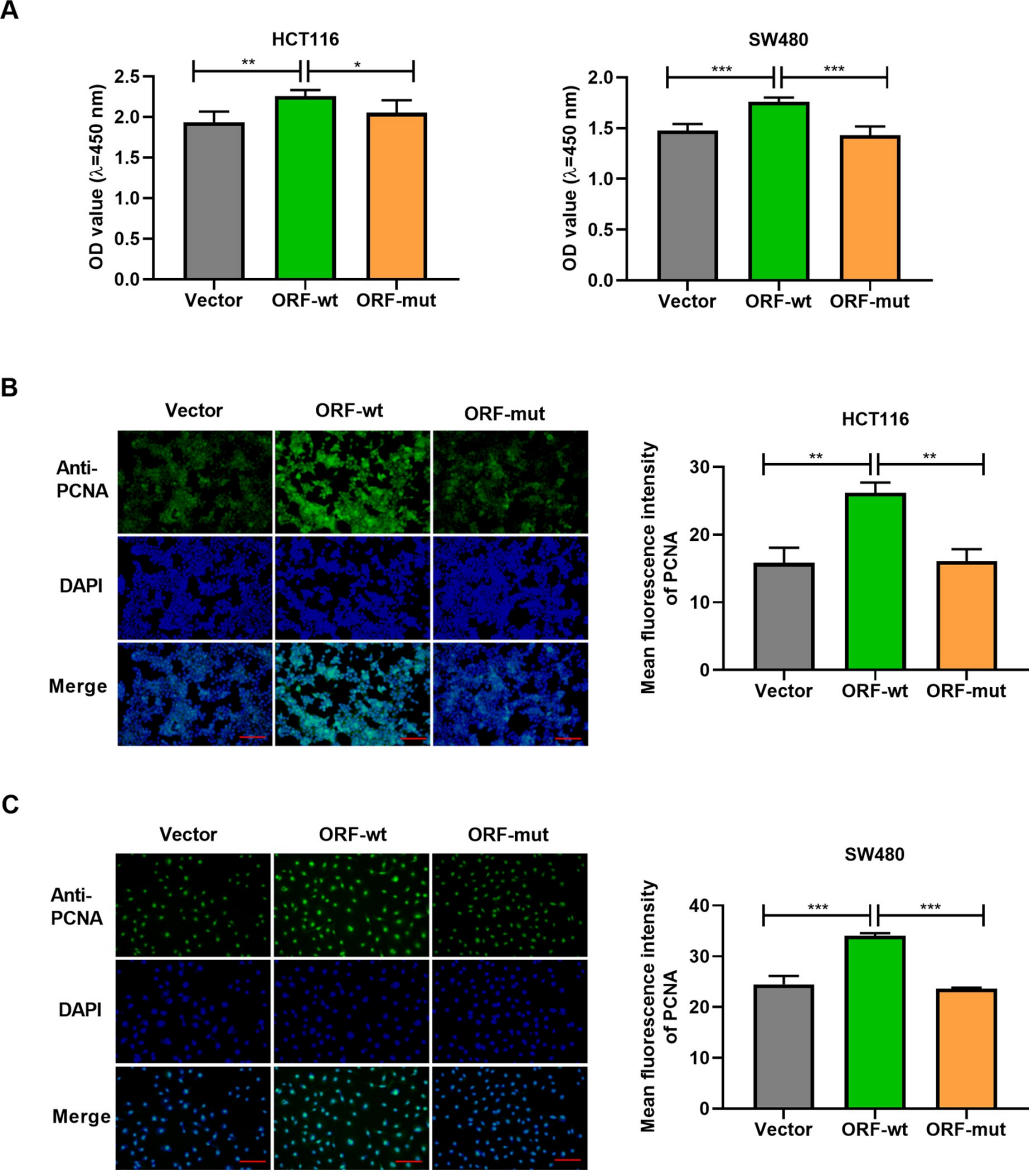
BVES-AS1-201-50aa promotes the viability of HCT116 and SW480 cells. (A-C) HCT116 and SW480 cells were introduced with an expression plasmid fused with 3× Flag tags (ORF-wt), the mutant expression plasmid (ORF-mut), or negative control plasmid (Vector). (A) CCK-8 cell viability assay was performed with the cells transfected as indicated. (B and C) Representative immunofluorescence images showed the expression of PCNA in cells after the indicated transfection. Scale Bar: 100 μm. *P<0.05, **P<0.01, ***P<0.001.

Next, we decided to study the effect of the peptide BVES-AS1-201-50aa on CRC cell malignant phenotypes, including cell growth, migration, and invasion. To address this, we expressed the peptide in HCT116 and SW480 cells using the expression plasmid fused with 3× Flag tags (ORFwt-Flag, also named ORF-wt). Strikingly, BVES-AS1-201-50aa expressed by ORF-wt increased the viability of HCT116 and SW480 cells compared with the vector control (Fig 2A). This result was also verified by staining for cell proliferation marker PCNA in ORF-wt-transfected cells. BVES-AS1-201-50aa caused a clear augmentation in PCNA expression in HCT116 (Fig 2B) and SW480 (Fig 2C) cells, measured through PCNA fluorescence intensity by immunofluorescence. To support these findings, the mutant expression plasmid (ORFmut-Flag, also called ORF-mut) was also transfected the two cell lines. Indeed, the mutation strongly abolished the effects of BVES-AS1-201-50aa on cell viability and PCNA expression (Fig 2A–2C).

**Fig 3:**
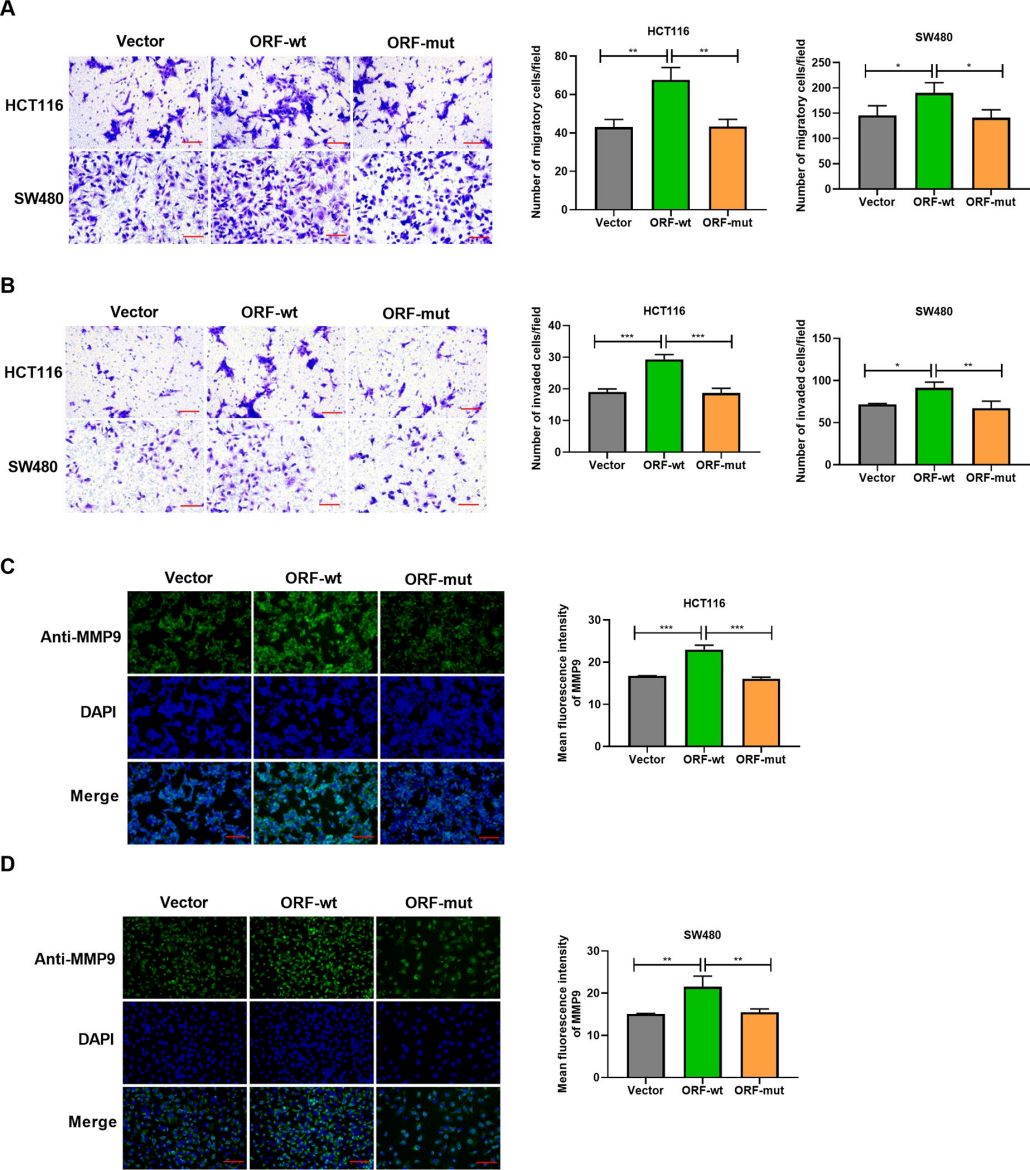
BVES-AS1-201-50aa enhances the migratory and invasive abilities of HCT116 and SW480 cells. (A-D) HCT116 and SW480 cells were introduced with an expression plasmid fused with 3× Flag tags (ORF-wt), the mutant expression plasmid (ORF-mut), or negative control plasmid (Vector). (A and B) Transwell motility and invasion assays were performed with the transfected cells. Scale Bar: 50 μm. (C and D) Representative immunofluorescence images showed MMP9 expression level in the cells transfected as indicated. Scale Bar: 100 μm. *P<0.05, **P<0.01, ***P<0.001.

Next, we asked whether BVES-AS1-201-50aa can affect the migratory and invasive behaviors of CRC cells by transwell assays. The results revealed that BVES-AS1-201-50aa expressed by ORF-wt remarkably enhanced the migratory and invasive abilities of HCT116 and SW480 CRC cells (Fig 3A and 3B). Furthermore, HCT116 and SW480 cells expressing BVES-AS1-201-50aa exhibited higher levels of metastasis-associated protein MMP9 than Vector-transfected cells (Fig 3C and 3D), validated via MMP9 fluorescence intensity. Importantly, introduction of ORF-mut abrogated BVES-AS1-201-50aa-caused promotion of motility and invasion of HCT116 and SW480 cells (Fig 3A and 3B). Additionally, immunofluorescence assay showed that ORF-mut transfection reduced MMP9 expression induced by BVES-AS1-201-50aa (Fig 3D). These findings together suggest that BVES-AS1-201-50aa can promote CRC cell growth, migration, and invasion *in vitro*.

### BVES-AS1-201-50aa targets the Src/mTOR signaling pathway in CRC cells

**Fig 4:**
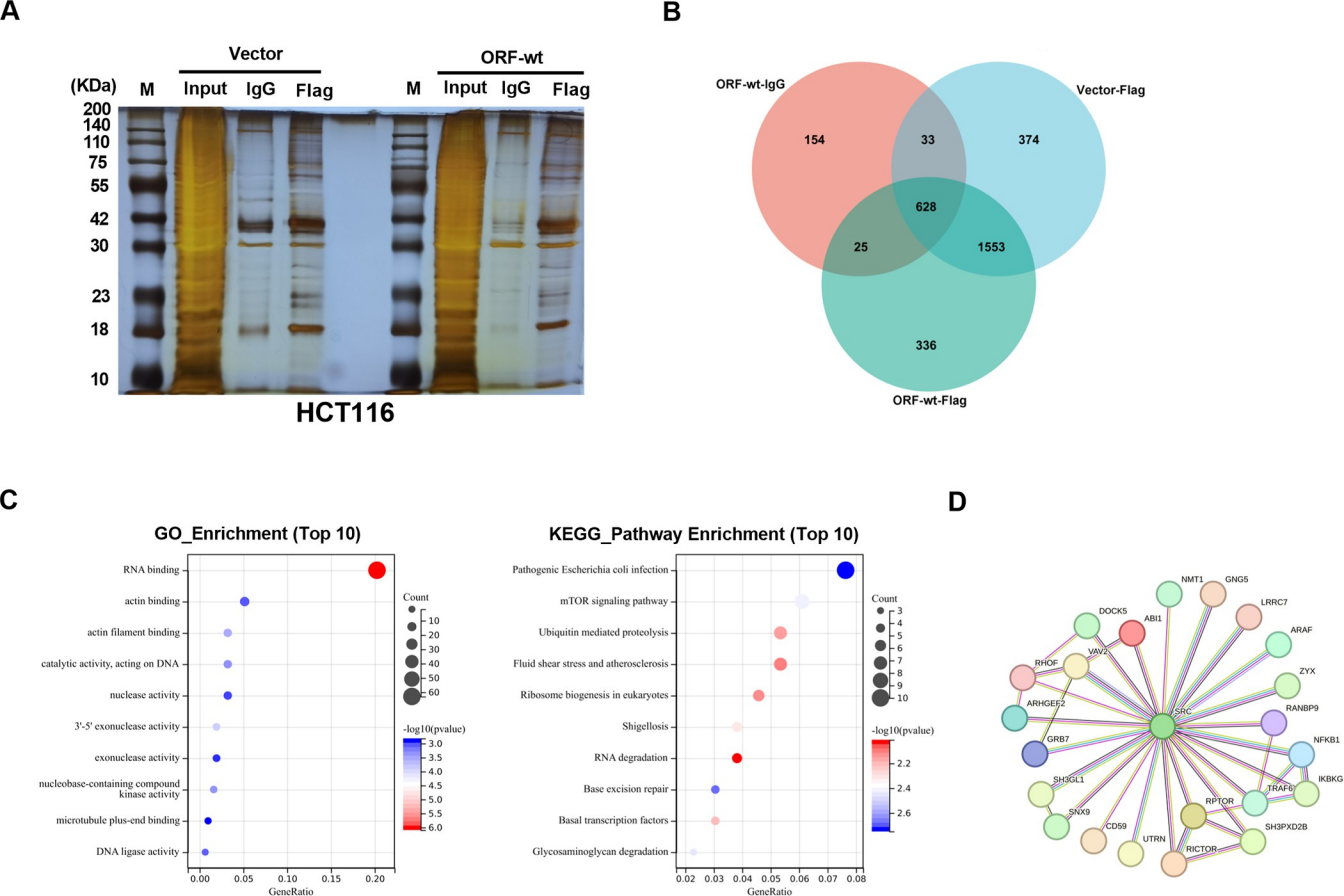
BVES-AS1-201-50aa interacts with the Src/mTOR pathway in CRC cells. (A) Vector- or ORF-wt-transfected HCT116 cells were lysed and incubated with beads-coupled anti-Flag antibody or isotype IgG. The precipitated proteins were isolated from the beads and subjected to SDS-PAGE electrophoresis before silver staining. (B) The precipitated proteins in the ORF-wt-IgG, Vector-Flag and ORF-wt-Flag groups were processed by qualitative proteomic analysis. Venn diagram showed the quantity of the precipitated proteins in the three groups and 336 unique proteins interacted with BVES-AS1-201-50aa. (C) The bubble plot revealed the top 10 enriched biological processes and the top 10 most enriched KEGG pathways of the 336 unique proteins interacted with BVES-AS1-201-50aa. (D) Schematic diagram of the interaction network of Src and 22 proteins.

To find potential target proteins affected by BVES-AS1-201-50aa, we performed qualitative proteomic analysis by a HPLC-MS/MS method after Co-IP experiments using beads-coupled anti-Flag antibody or isotype IgG control in ORF-wt-transfected HCT116 cells. Meantime, we adopted the silver staining technique of SDS polyacrylamide gels of the precipitated proteins to analyze the binding efficiency of anti-Flag antibody-coupled beads. The results showed that many proteins might be pulled down by the anti-Flag antibody in the ORF-wt group (Fig 4A). To rule out protein impurity, the precipitated proteins in the Vector-Flag and ORF-wt-IgG control groups were also processed by qualitative proteomic analysis. After the data of the two control groups to rule out protein impurity, a total of 336 unique proteins were pulled down by the anti-Flag antibody in the ORF-wt-transfected HCT116 cells (Fig 4B and S2 Table). Because BVES-AS1-201-50aa-Flag fusion peptide could be pulled down by anti-Flag antibody and the Vector-Flag control group could be used to eliminate proteins related to Flag tag, these results suggested that BVES-AS1-201-50aa peptide could specifically interact with 336 proteins in HCT116 CRC cells.

To observe the related biological process and signaling pathways of the 336 proteins, we performed GO enrichment analysis and KEGG pathway enrichment analysis on DAVID database. GO enrichment analysis showed that these proteins had a close relationship with ribosome functions, including RNA binding, nuclease activity and nuclease-containing compound kinase activity (Fig 4C). The ability to interact with ribosomes is the essential condition for lncRNA to have coding potential [25]. Thus, these data supported the coding activity of the BVES-AS1-201-50aa ORF. Intriguingly, via KEGG pathway enrichment analysis, the mTOR signaling pathway was found to be one of the most prominent enriched pathways (Fig 4C), which possesses a pivotal function in colorectal tumorigenesis [26]. We further analyzed the protein-protein interaction (PPI) of the 336 proteins on the online tool String (https://cn.string-db.org/) and found a complex regulatory network (S1 Fig). More interestingly, Src, acting as an upstream mediator of the mTOR signaling [27, 28], was the network hub of a significant PPI sub-network (Fig 4D). Among the 22 proteins interacted with Src, several proteins, such as RPTOR and TRAF6, were found to be closely related to the mTOR signaling pathway (Fig 4D). These data suggest that BVES-AS1-201-50aa may interact with the Src/mTOR signaling pathway in CRC cells.

### BVES-AS1-201-50aa activates the Src/mTOR signaling pathway in CRC cells

**Fig 5:**
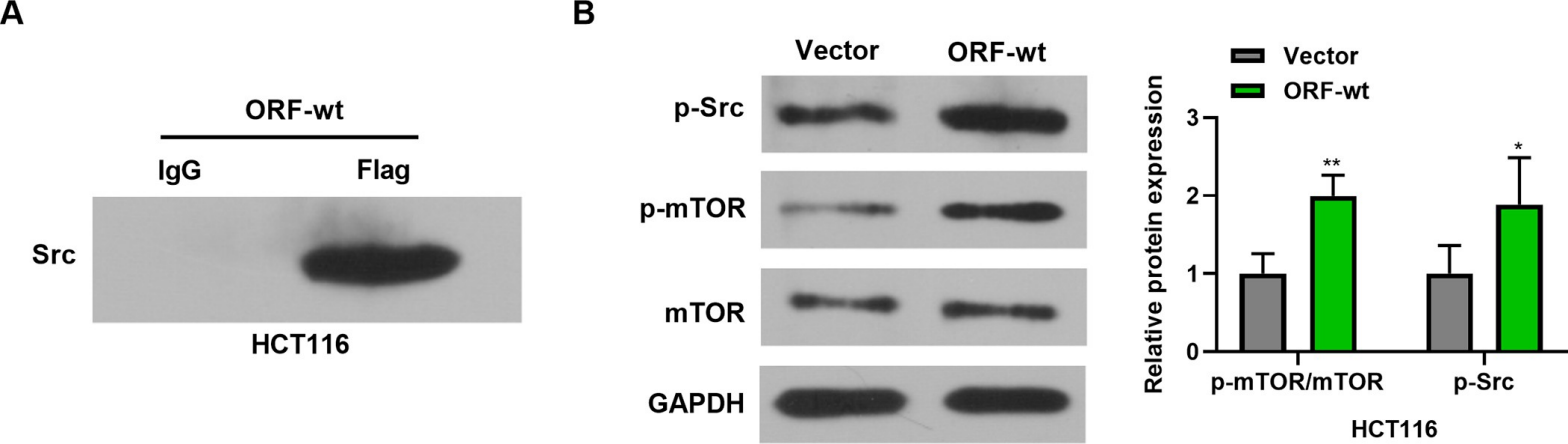
BVES-AS1-201-50aa promotes the activation of the Src/mTOR pathway in CRC cells. (A) ORF-wt-transfected HCT116 cells were subjected to Co-IP experiments using an anti-Flag antibody or isotype IgG, and the enrichment level of Src in the immunoprecipitates was confirmed by western blotting with the anti-Src antibody. (B) Western blotting showed the levels of p-Src, p-mTOR, and mTOR in HCT116 cells after transfection by ORF-wt or Vector control, with GAPDH as a loading control for expression quantification. *P<0.05, **P<0.01.

Based on the above findings, we further studied how BVES-AS1-201-50aa affects the Src/mTOR pathway in CRC cells. To resolve this, ORF-wt plasmid was transfected into HCT116 CRC cells and subsequently Co-IP experiments were performed using the anti-Flag antibody, followed by the acquisition of beads-bound proteins. The enrichment levels of Src in the pull-down proteins were further measured by western blotting using the antibody against Src. The results revealed that in HCT116 cells expressing BVES-AS1-201-50aa-Flag fusion peptide, Src protein was drastically enriched by the anti-Flag antibody compared to IgG control (Fig 5A), indicating that BVES-AS1-201-50aa could bind Src in HCT116 CRC cells. Furthermore, BVES-AS1-201-50aa expressed by ORF-wt induced the activation of the Src/mTOR pathway, as presented by the increase of p-Src level and p-mTOR/mTOR ratio in HCT116 cells (Fig 5B). Taken together, these results indicate that BVES-AS1-201-50aa enhances the activation of the Src/mTOR pathway in CRC cells.

## Discussion

Many lncRNAs as the noncoding transcripts have been extensively explored and ascribed crucial functions in human tumorigenesis [29]. Lately, the advancement of bioinformatic and biochemical methodologies has revealed that a significant proportion of lncRNAs encompass short ORFs that have protein-encoding potential [11]. Moreover, lncRNA-encoded peptides have emerged as critical regulators in the development of various cancers, including CRC [12, 13, 16]. Herein, we have demonstrated that lncRNA BVES-AS1 can encode a 50-aa short peptide, named BVES-AS1-201-50aa. This peptide can promote CRC cell viability and enhance cell migratory and invasive capacities *in vitro*, implying that BVES-AS1-201-50aa may function as a tumor driver in CRC. PCNA is essential for DNA replication [30], and MMP9 is related to cancer cell metastasis [31], both of which contribute to CRC development [32, 33]. In this study, the expression alteration of PCNA and MMP9 also support the oncogenic role of BVES-AS1-201-50aa in CRC. Our study broadens the diversity and breadth of lncRNAs in human tumorigenesis, with the hope that such peptides may have an application value for the identification of tumor biomarkers and the development of anti-tumor drugs in the future.

Previous work proved that lncRNA BVES-AS1 can operate as a tumor suppressor in CRC by repressing colon adenocarcinoma cell growth, motility, and metastasis through the miR-522-3p/BVES cascade [34]. Our current study suggests the tumor-promoting activity of BVES-AS1-201-50aa in CRC, indicating that BVES-AS1 and its peptide BVES-AS1-201-50aa have opposite effects in regulating colorectal tumorigenesis. Similarly, although LINC00665 functions as a pro-tumor factor in breast cancer (BC) [35, 36], its small peptide CIP2A-BP can lead to suppressed abilities of migration and invasiveness of BC cells [35]. On the other hand, Li *et al*. uncovered that both LINC00665 and its micro-peptide CIP2A-BP contribute to the progression of hepatocellular carcinoma by enhancing cancer cell growth and motility [37]. These findings suggest that lncRNAs and their micro-peptides may exert consistent or contradictory functions in cancer cells, and the same peptide may play a contradictory role in different types of tumors.

Surprisingly and intriguingly, through qualitative proteomic analysis after Co-IP experiments, we found that BVES-AS1-201-50aa may interact with Src in CRC cells. The tyrosine kinase Src is dysregulated in more than 80% of CRC cases, and increased expression of Src is related to a poor prognosis and can induce CRC cell growth, invasion, and drug resistance [19, 20, 38]. According to the KEGG pathway enrichment analysis and PPI analysis, we further found the relationship between BVES-AS1-201-50aa and the Src/mTOR signaling pathway, which is a key pathway in cancer biology and actively participates in the pathogenesis of multiple cancers, such as melanoma, hepatocellular carcinoma and non-small cell lung cancer [27, 28, 39]. Importantly, we first demonstrate that BVES-AS1-201-50aa can activate the Src/mTOR signaling pathway in CRC cells. The activation of the mTOR signaling pathway can strongly promote colorectal carcinogenesis [40, 41]. With these findings, we conclude that BVES-AS1-201-50aa contributes to CRC pathogenesis by activating the Src/mTOR pathway. The current study is hampered by the lack of *in vivo* research using the animal experimental models with orthotopic CRC tumors or xenograft tumors, which should be performed in the future.

## Conclusions

In this study, our data established the novel notion that lncRNA BVES-AS1 could encode a micro-peptide BVES-AS1-201-50aa. BVES-AS1-201-50aa could promote cell growth and enhance migratory and invasive capacities of CRC cells *in vitro*. Furthermore, BVES-AS1-201-50aa enhanced the activation of the Src/mTOR signaling pathway. Taken together, BVES-AS1-201-50aa could function as a driver of CRC cell growth, migration, and invasion. Therefore, developing pharmacological agents that target BVES-AS1-201-50aa may have a novel opportunity for CRC treatment.

## Supporting information

S1 Fig The complex regulatory network of the 336 unique proteins interacted with BVES-AS1-201-50aa. (TIF)

S1 Table The detailed information of seven peptides encoded by three lncRNAs. (XLSX)

S2 Table The 336 unique proteins pulled down by the anti-Flag antibody in the ORF-wt-transfected HCT116 cells. (XLSX)

S1 Raw images (PDF)
