# Supplementary figures and images for: Peptide encoded by lncRNA BVES-AS1 promotes cell viability, migration, and invasion in colorectal cancer cells via the SRC/mTOR signaling pathway
Source: PLoS One. 2023 Jun 22;18(6):e0287133. doi: 10.1371/journal.pone.0287133 (PMC10286995; doi:10.1371/journal.pone.0287133)

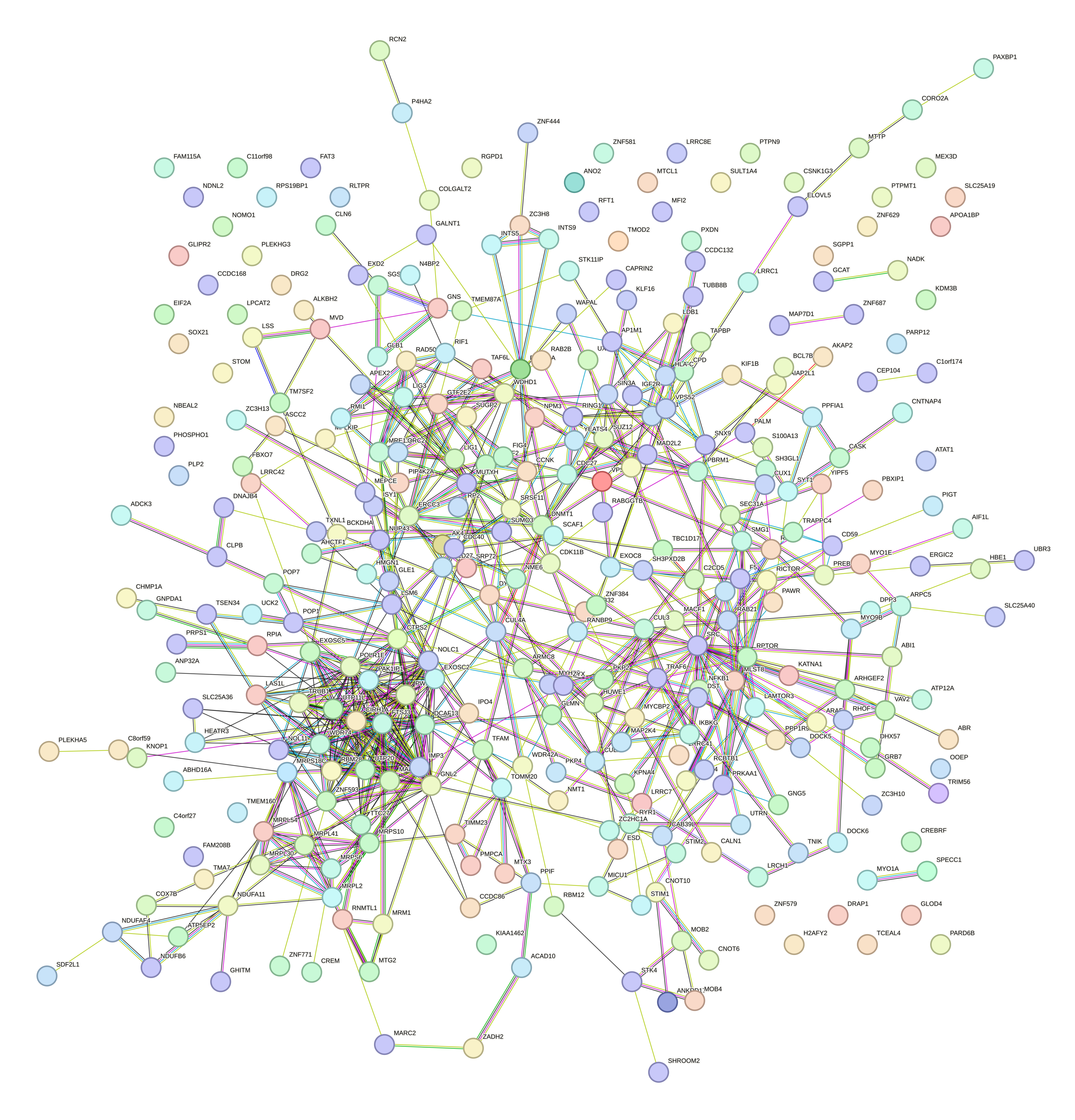

Supplement: S1 Fig — (TIF) [file pone.0287133.s001.tif]
