# Supplementary material for: Peptide encoded by lncRNA BVES-AS1 promotes cell viability, migration, and invasion in colorectal cancer cells via the SRC/mTOR signaling pathway
Source: PLoS One. 2023 Jun 22;18(6):e0287133. doi: 10.1371/journal.pone.0287133 (PMC10286995; doi:10.1371/journal.pone.0287133)
Supplement: S1 Raw images — (PDF) [file pone.0287133.s004.pdf]

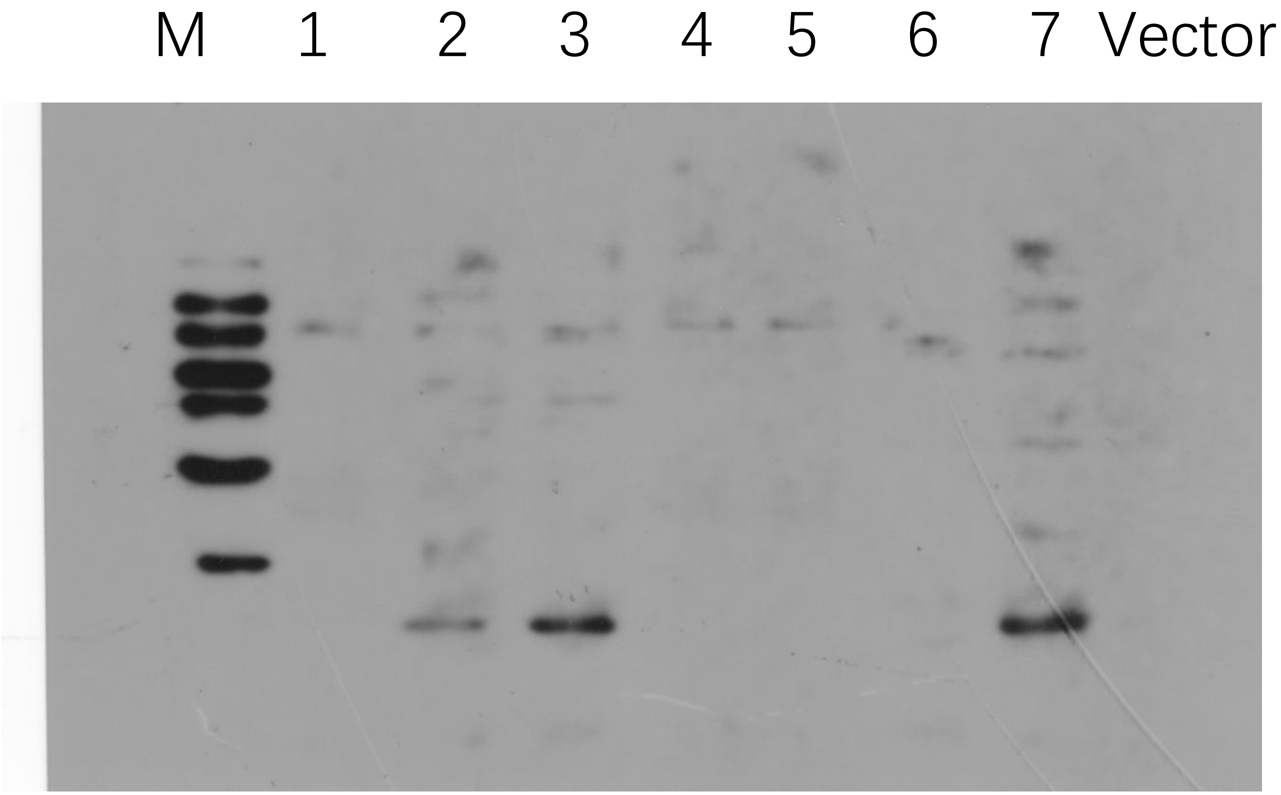
M 1 2 3 4 5 6 7 Vector

Fig.1B

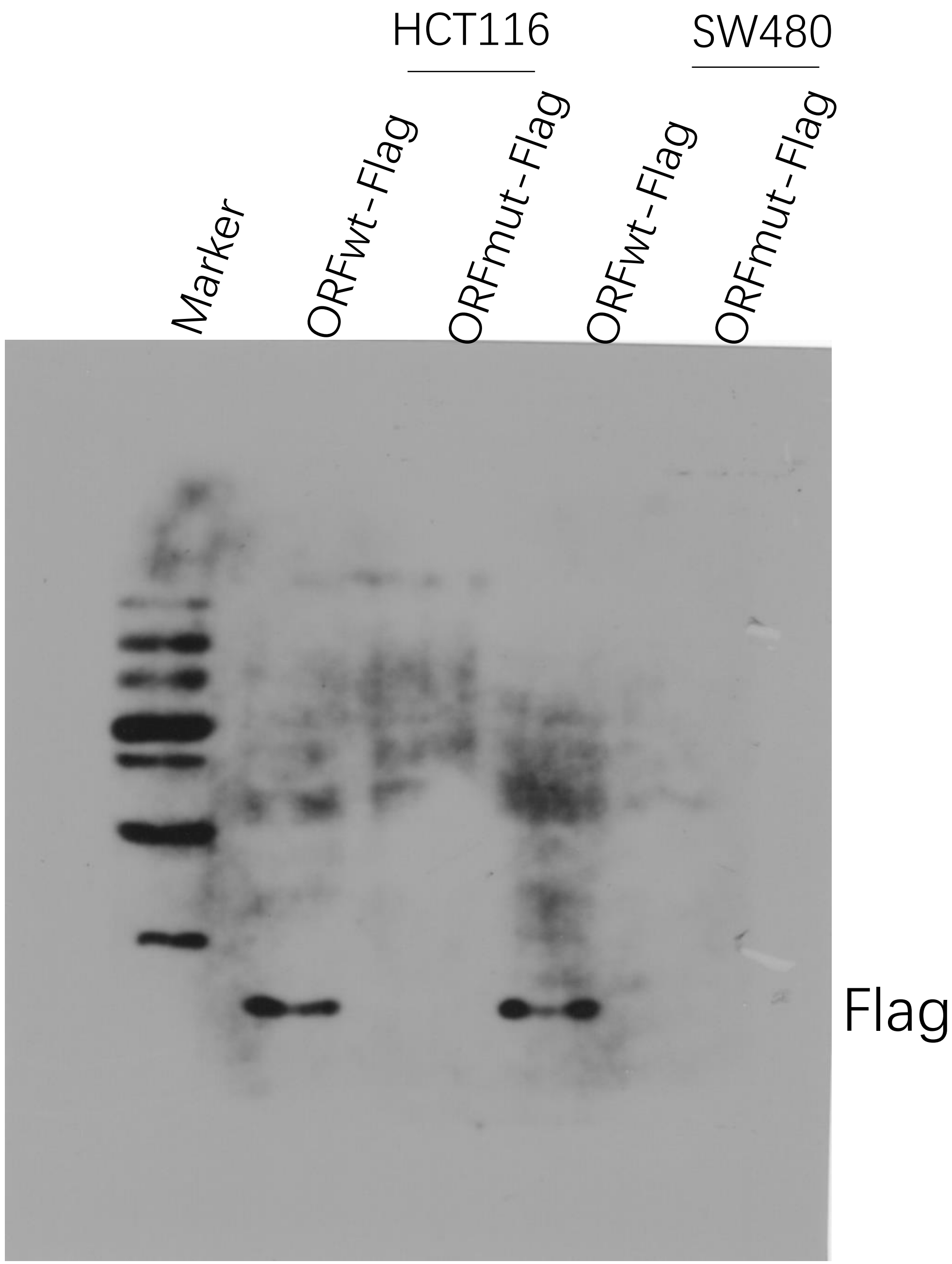
HCT116
SW480
Marker
ORFwt-Flag
ORFmut-Flag
ORFwt-Flag
ORFmut-Flag
Flag

Fig.1E

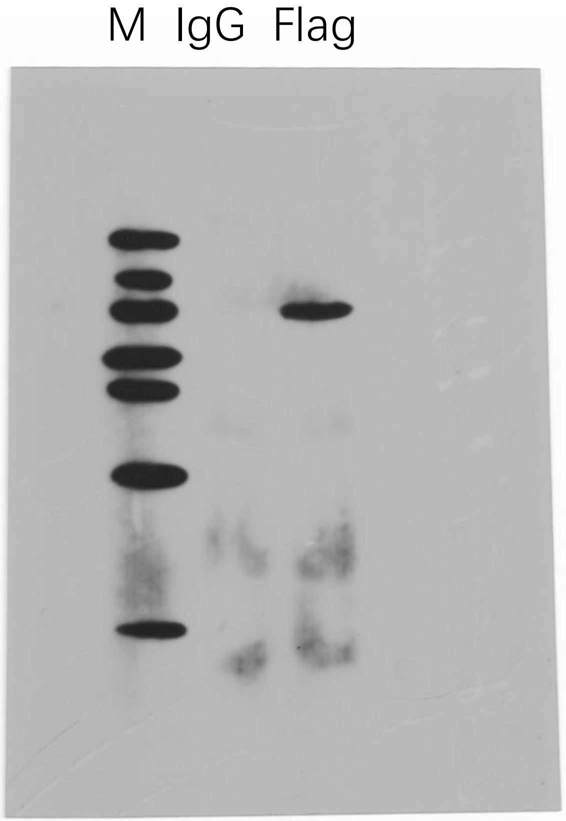

Fig.5A

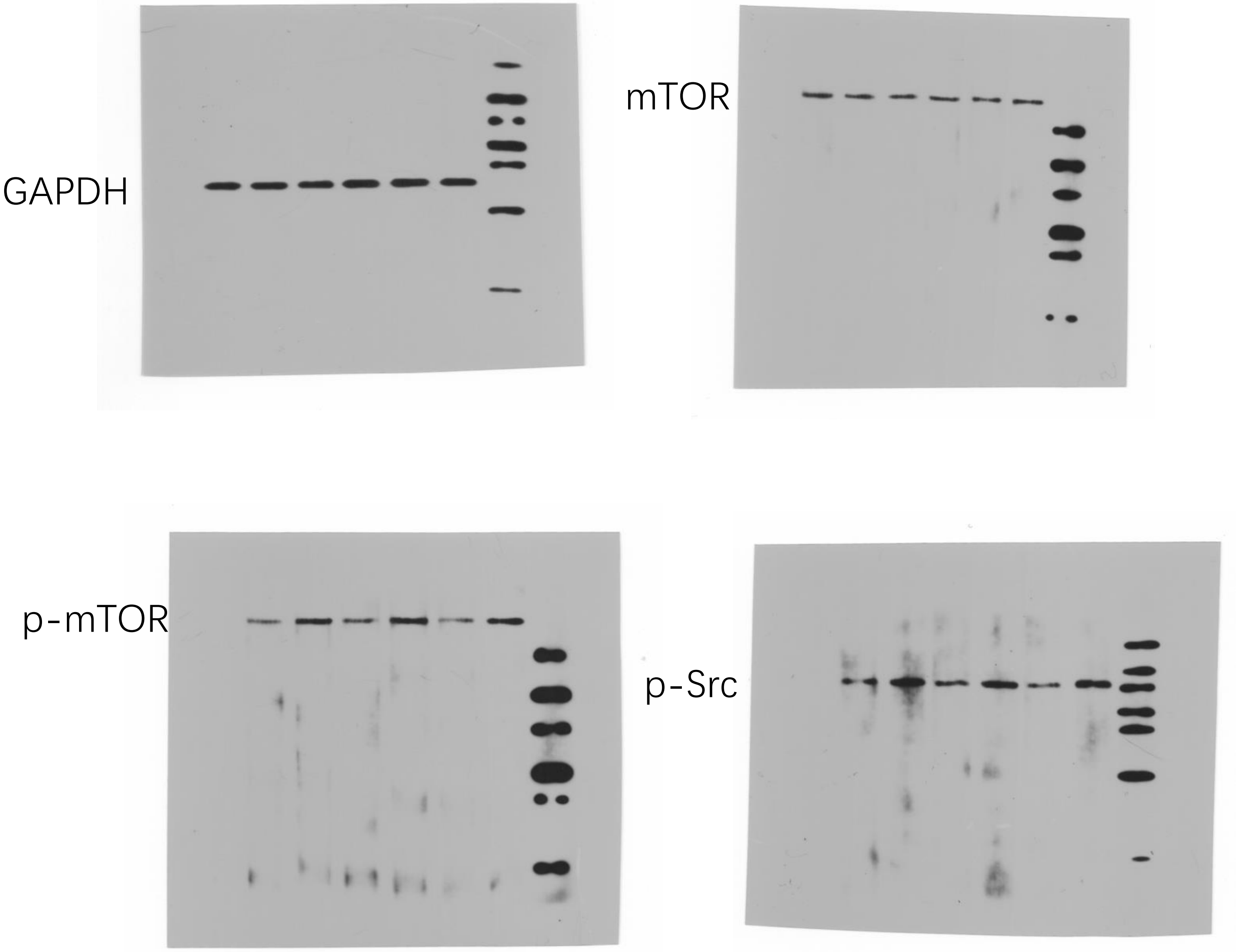

Fig.5B (lanes from left to right: Vector, ORF-wt, Vector, ORF-wt, Vector, ORF-wt, Marker)

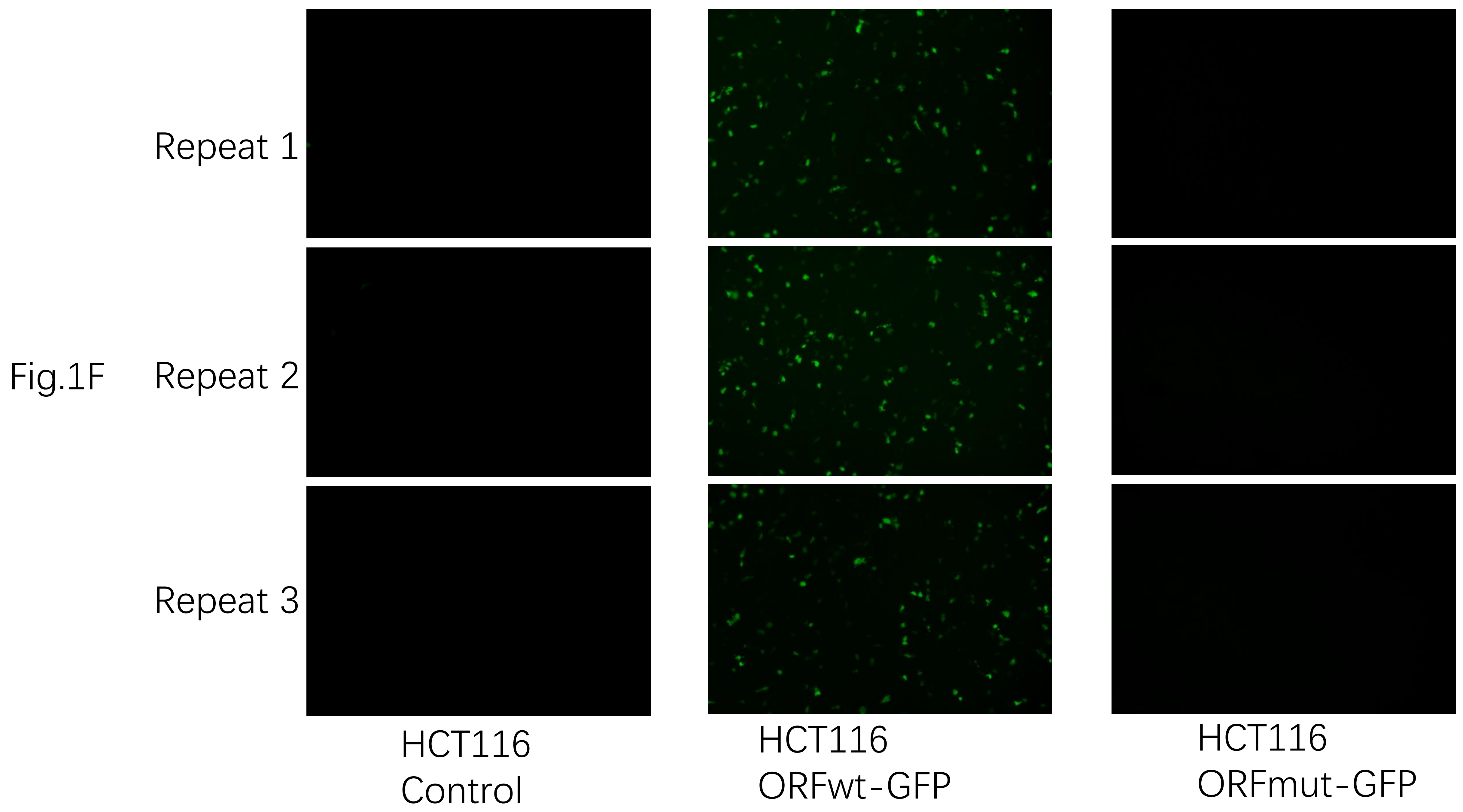
Fig.1F
Repeat 1
Repeat 2
Repeat 3
HCT116
Control
HCT116
ORFwt-GFP
HCT116
ORFmut-GFP

Fig.1F

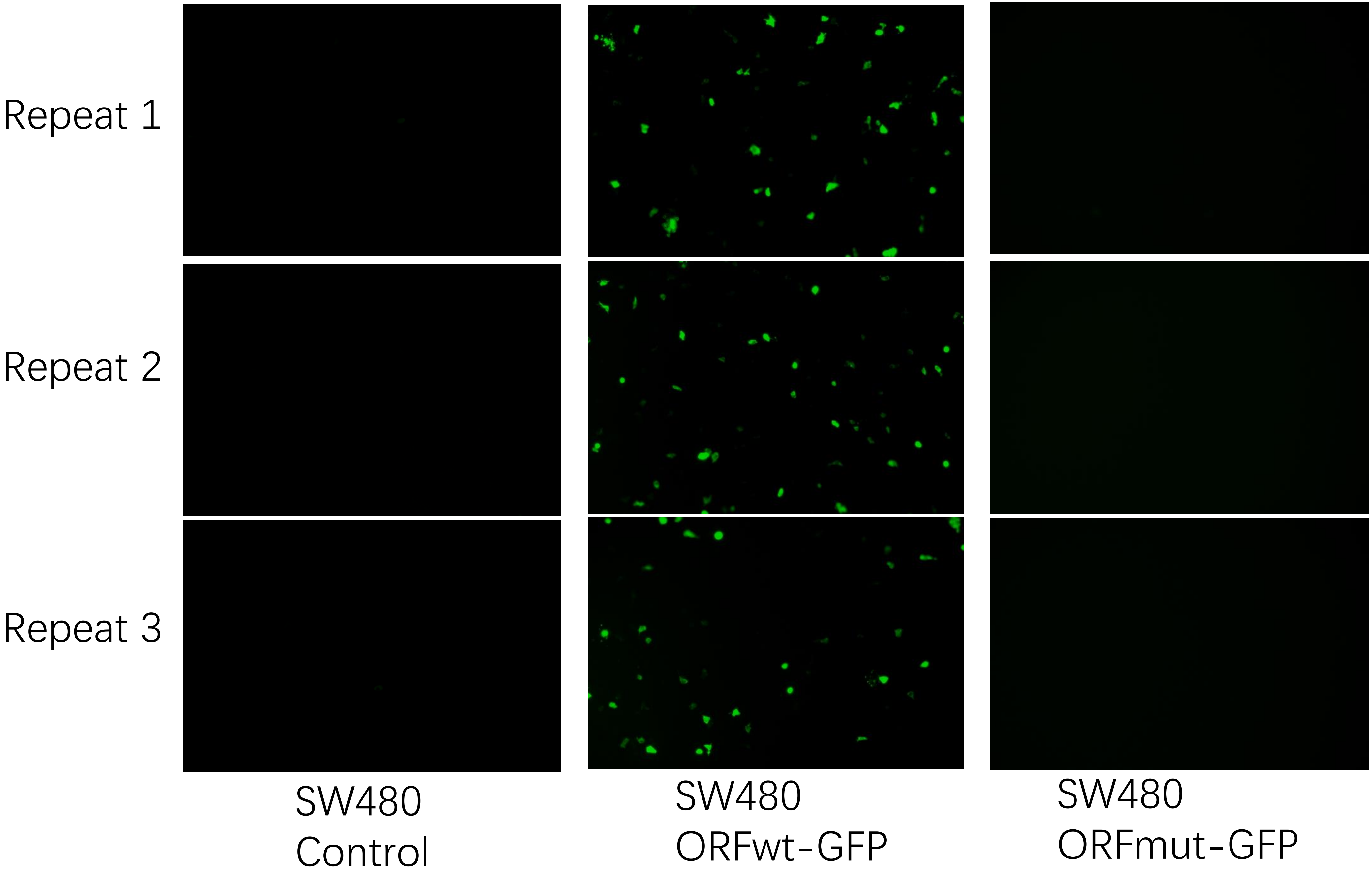
Repeat 1
Repeat 2
Repeat 3
SW480
Control
SW480
ORFwt-GFP
SW480
ORFmut-GFP

Fig.2B （three repeats） HCT116

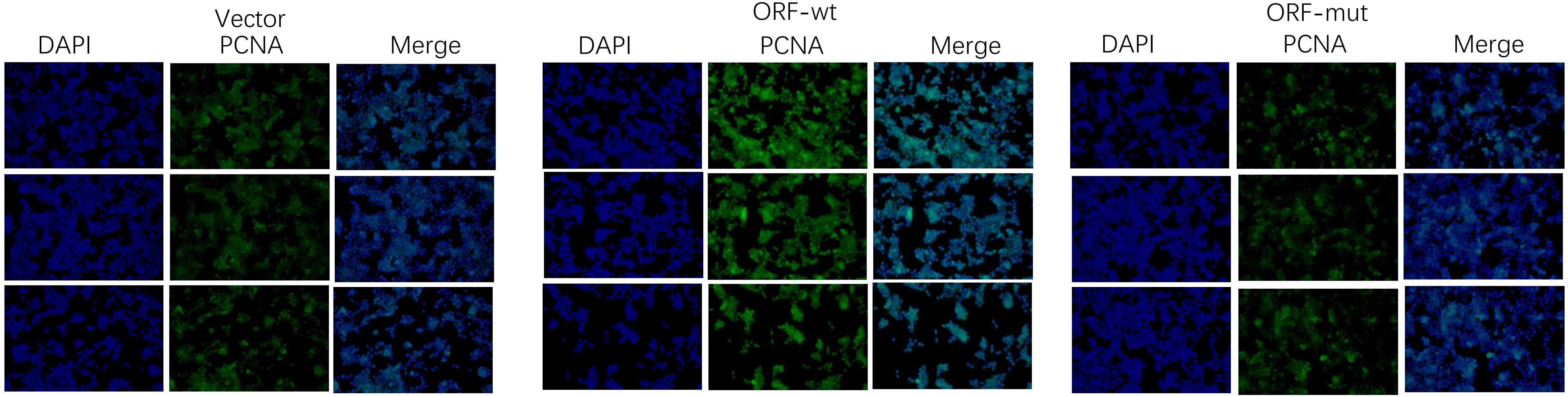

Fig.2C （three repeats） SW480

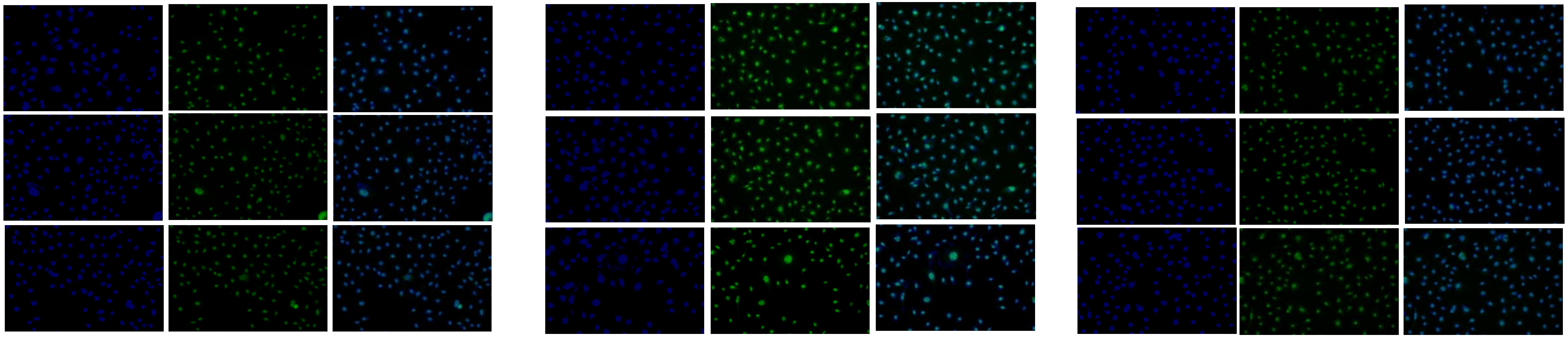

Fig.3A
(three
repeats)
Migration

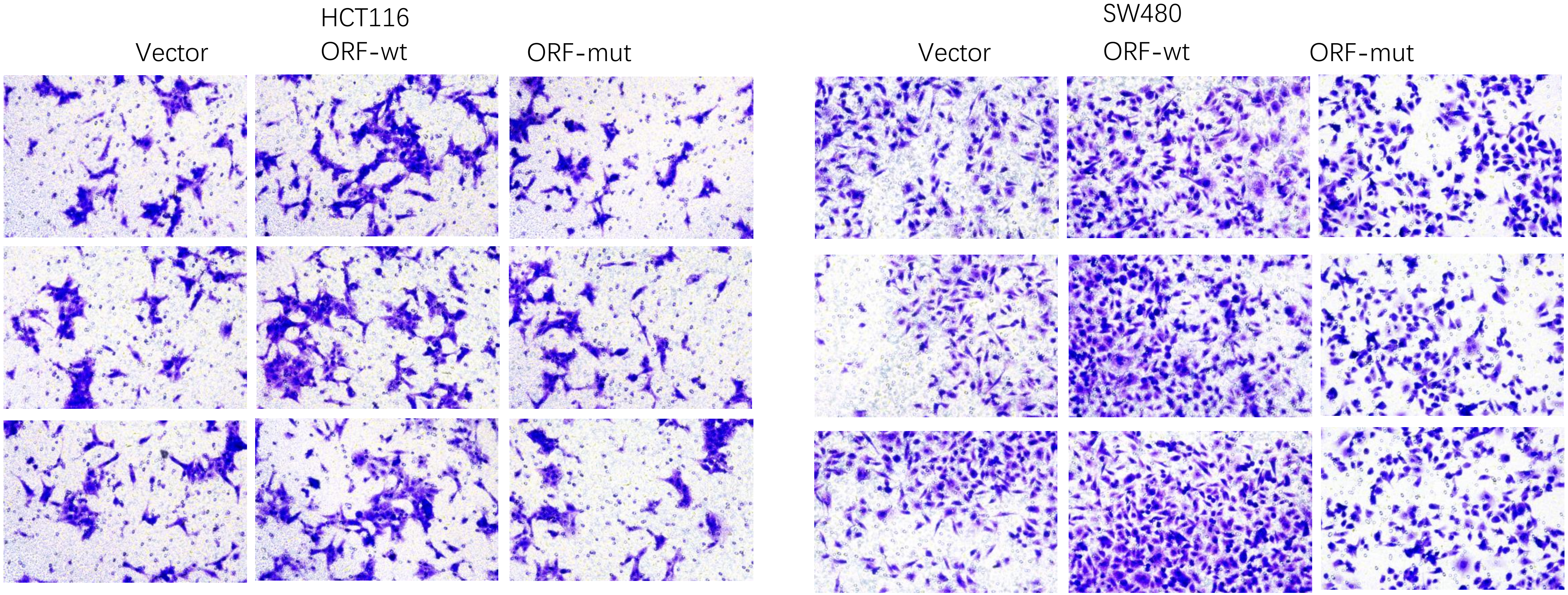

Fig.3B (three repeats) Invasion

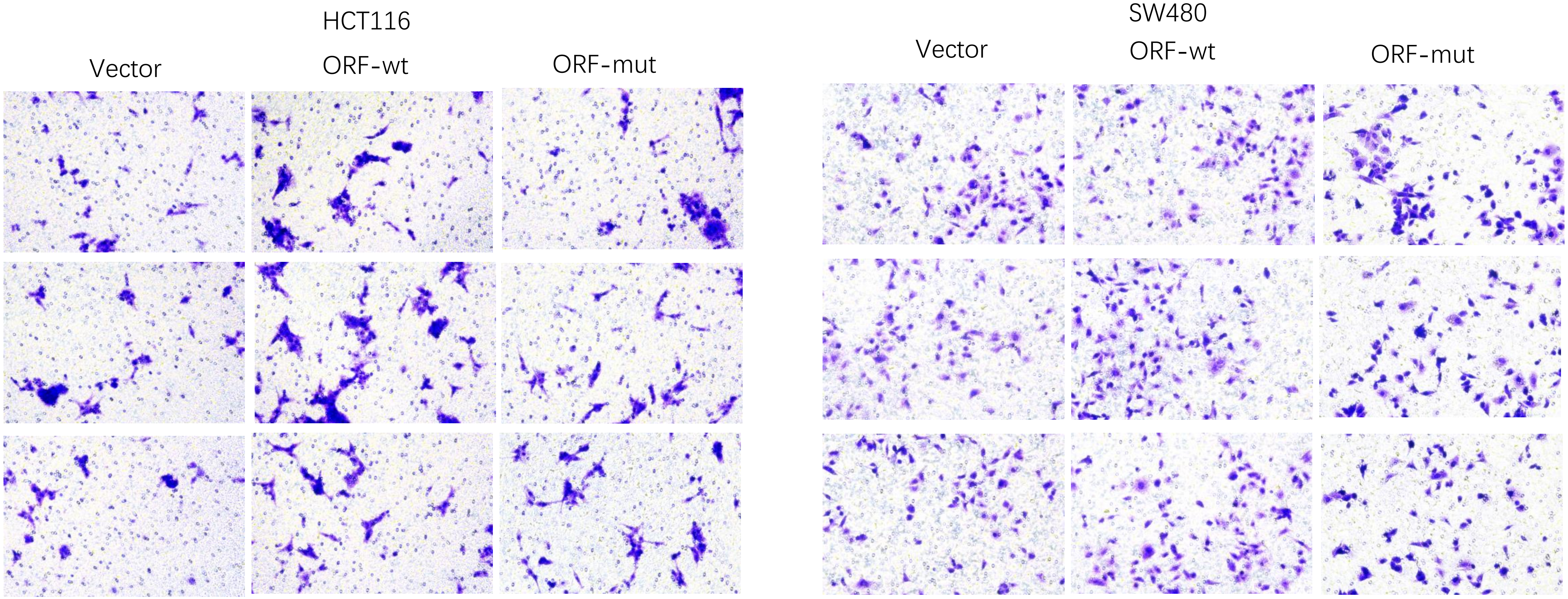

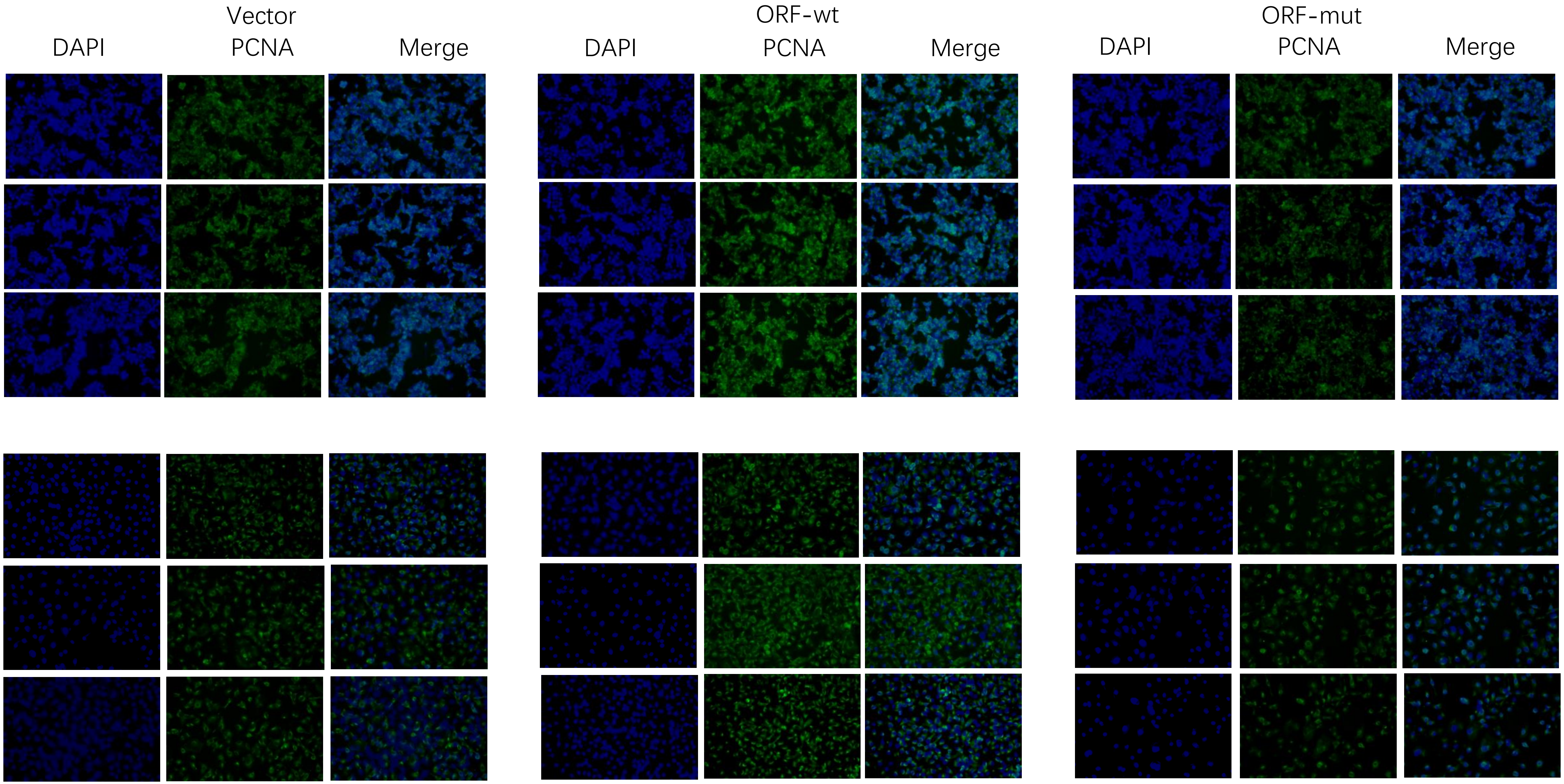

Fig.3C (three repeats) HCT116

Fig.3D (three repeats) SW480
